# A New Role Discovered for a Well-Known Clock Protein

**DOI:** 10.1371/journal.pbio.1002294

**Published:** 2015-11-12

**Authors:** Richard Robinson

**Affiliations:** Freelance Science Writer, Sherborn, Massachusetts, United States of America

## Abstract

A new study adds further complexity to the mammalian circadian clock by revealing that the CRY protein has an additional unsuspected feedback role in facilitating a crucial regulatory phosphorylation event. Read the Research Article.

Virtually any periodic phenomenon can function as a clock, from the swing of a pendulum to the surge of the tides. Within the cells of the brain’s suprachiasmatic nucleus, the body’s circadian clock is a complex and remarkably well-characterized set of proteins that control one another’s creation, activity, and destruction with temporal regularity, in turn driving the daily oscillation in expression of about 10% of our genes.

While much of what makes the cellular clock tick has been mapped in detail, other parts remain unknown. A central clock protein is BMAL1, whose periodic phosphorylation by casein kinase (CK2) is a critical event preceding BMAL1’s entrance into the nucleus, where it helps regulate those many genes. But CK2 by itself is thought to be constitutively active; so what entrains its activity to the daily rhythms of the cellular clock? In a new study, Teruya Tamaru and colleagues discover the answer in a previously unknown role played by another central clock protein, called CRY.

BMAL1 is phosphorylated by the enzymatically active subunit of CK2, called CK2α, a process that is inhibited by a second subunit, CK2β (Fig 1). The authors showed that BMAL1 and CK2β directly interact, but that interaction was reduced in the absence of CRY. The possibility that CRY might be involved in regulating BMAL1 phosphorylation had been raised by previous work, which showed that deficiency of CRY leads to hyperphosphorylation of BMAL1. Together, these results led Tamaru and colleagues to further pursue CRY’s role in regulating BMAL phosphorylation.

**Fig 1:**
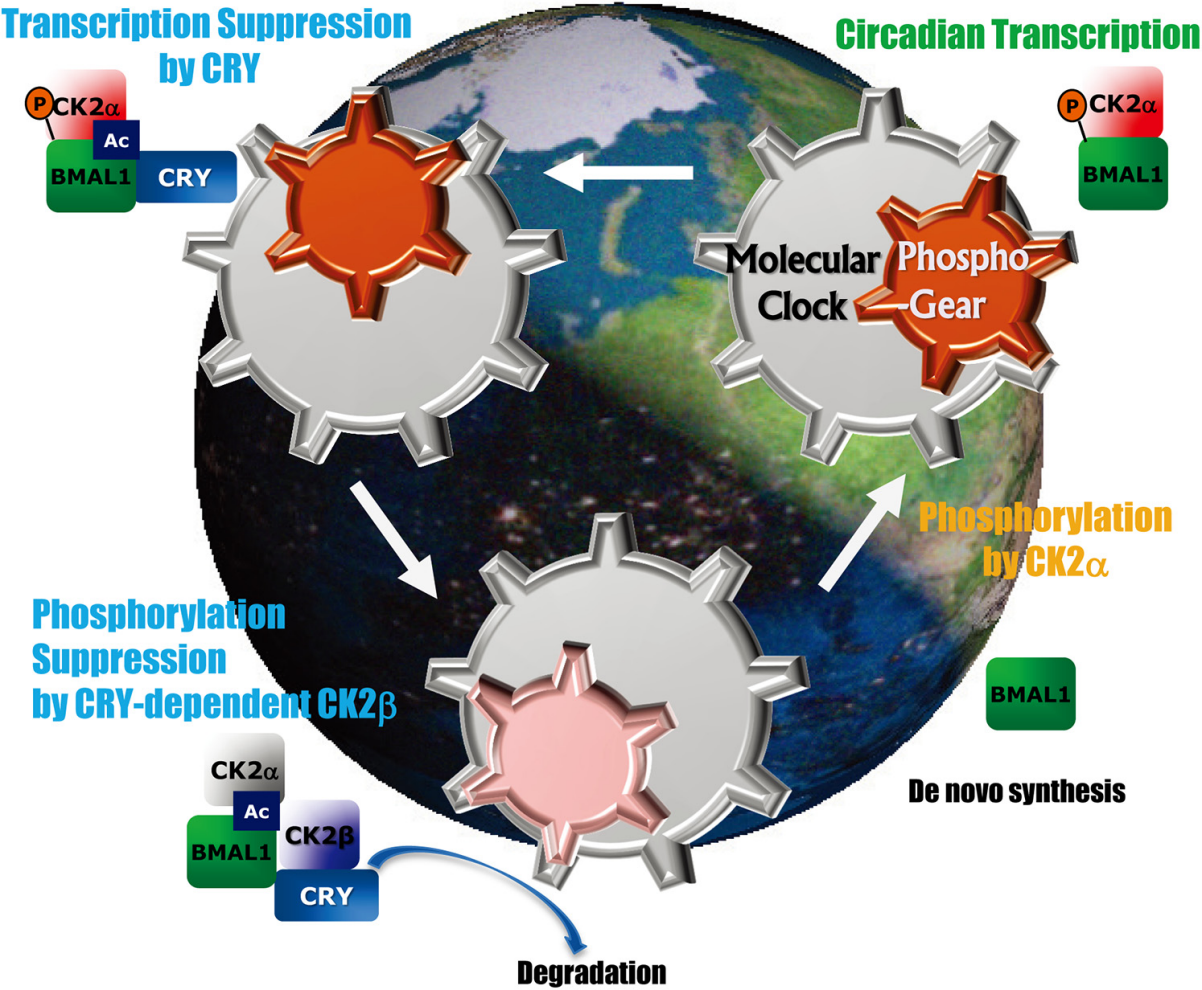
A “phospho-geared” circadian clock in mammals. CRY-driven cyclic phosphorylation of BMAL1 by CK2 serves as an integral gear in the mammalian clockwork that drives daily rhythms in physiology and behavior. *Image credit*: *Teruya Tamaru*.

If CRY is regulating the interaction of CK2β and BMAL1, does it bind to one or the other separately, or only to the pair? By knocking out BMAL1, the authors showed that it was not necessary for CRY-CK2β interaction, suggesting a sequential binding process, in which CRY first links with CK2β, followed by interaction with BMAL1. This model implied that oscillation in CRY was driving the oscillation in BMAL phosphorylation.

In support of that hypothesis, the authors found that knocking out CRY led to loss of periodic suppression of phosphorylation, and thus constitutively high levels of phosphorylated BMAL1; restoring CRY rescued this effect. Furthermore, CRY proteins co-precipitated with BMAL1 (or with CK2β alone) in a circadian rhythm aligned with the interaction of BMAL1 and CK2β, suggesting that it was the appearance of CRY that triggered the interaction. Further support came from an assay in live cells, in which complementary halves of a bioluminescence enzyme were attached to CK2β and BMAL1. Overexpression of CRY led to enhanced bioluminescence, indicating an increased association of CK2β and BMAL1, in time with the circadian rhythm of the living system. Thus, the authors argue, the cyclic phosphorylation of BMAL1 is due to oscillatory inhibition driven by CRY (Fig 1). The authors also identified critical regions on BMAL1 and CRY for CRY-enhanced binding of CK2β to BMAL1.

Finally, they showed that the phosphorylation event catalyzed by CK2α and regulated by CK2β was necessary for a subsequent acetylation of BMAL1 (Fig 1). That acetylation was, in turn, required for the recruitment of CRY/CK2β, preventing further phosphorylation. Dephosphorylation of BMAL1 returns it to the start of the cycle, from which it can be phosphorylated again, enter the nucleus, and play its part as a transcription factor.

As befits a mechanism responsible for regulating so many genes, the workings of the cellular clock are complex. The feedback system identified in this study forms one loop within a system containing several more, designed to robustly and flexibly align the rhythms of the organism to the rhythms of the environment. In multiple human disorders, those two sets of rhythms can misalign, making understanding of the clock potentially important in designing therapies to bring the body back in rhythm with its environment.
